# Post-Stroke Working Memory Dysfunction: A Meta-Analysis and Systematic Review

**DOI:** 10.1007/s11065-020-09462-4

**Published:** 2020-11-24

**Authors:** Selma Lugtmeijer, Nikki A. Lammers, Edward H. F. de Haan, Frank-Erik de Leeuw, Roy P. C. Kessels

**Affiliations:** 1 University of Amsterdam, Amsterdam, the Netherlands; 2 Radboud University Medical Center, Department of Neurology, Nijmegen, the Netherlands; 3 Donders Institute for Brain, Cognition and Behaviour, Radboud University, Nijmegen, the Netherlands; 4 Department of Medical Psychology, Radboud University Medical Center, Nijmegen, the Netherlands

**Keywords:** Stroke, Cognition, Working memory, Prevalence, Systematic review, Meta-analysis

## Abstract

This review investigates the severity and nature of post-stroke working memory deficits with reference to the multi-component model of working memory. We conducted a systematic search in PubMed up to March 2019 with search terms for stroke and memory. Studies on adult stroke patients, that included a control group, and assessed working memory function, were selected. Effect sizes (Hedges’ *g*) were extracted from 50 studies (in total 3,084 stroke patients) based on the sample size, mean and standard deviation of patients and controls. Performance of stroke patients was compared to healthy controls on low-load (i.e. capacity) and high-load (executively demanding) working memory tasks, grouped by modality (verbal, non-verbal). A separate analysis compared patients in the sub-acute and the chronic stage. Longitudinal studies and effects of lesion location were systematically reviewed. Stroke patients demonstrated significant deficits in working memory with a moderate effect size for both low-load (Hedges’ *g* = -.58 [-.82 to -.43]) and high-load (Hedges’ *g* = -.59 [-.73 to -.45]) tasks. The effect sizes were comparable for verbal and non-verbal material. Systematically reviewing the literature showed that working memory deficits remain prominent in the chronic stage of stroke. Lesions in a widespread fronto-parietal network are associated with working memory deficits. Stroke patients show decrements of moderate magnitude in all subsystems of working memory. This review clearly demonstrates the global nature of the impairment in working memory post-stroke.

## Introduction

Stroke survivors may be challenged not only with physical disability, but also with cognitive consequences. Dysfunction in perception, executive functioning, abstract reasoning, episodic memory or language has been found to be present in 60–70% of stroke patients (Nys, Van Zandvoort, Worp, Kappelle, & Haan, 2007; De Haan, Nys, & Van Zandvoort, 2006). Post-stroke cognitive impairment has been associated with functional dependency (e.g. Saxena, Ng, Koh, Yong, & Fong, 2007) and poorer quality of life (e.g. Mellon, Brewer, Hall, Horgan, Williams, & Hickey, 2015). Whereas post-stroke dementia and episodic memory function after stroke have been abundantly studied and reviewed (see, for instance, reviews by Pendlebury & Rothwell, 2009, and Lim & Alexander, 2009, respectively), a comprehensive overview of working memory deficits as a consequence of stroke is lacking. This is striking, as working memory/updating is considered to be one of the key subdomains of executive function along with shifting and inhibition (see e.g. comprehensive reviews by Chan, Shum, Toulopoulou, & Chen, 2008 and Friedman & Myake, 2017). Deficits in this memory system may not only affect other executive functions, but also episodic memory formation and retrieval (Bergmann, Kiemeneij, Fernández, & Kessels, 2013). Furthermore, a recent prospective cohort study on cognitive function and long-term functional outcome after stroke at a young age (< 50) showed that only decline in working memory predicted poor functional outcome 11 years after the stroke (Synhaeve, Schaapsmeerders, Arntz, Maaijwee, Rutten-Jacobs, Schoonderwaldt, & de Leeuw, 2015).

Working memory is generally thought of as a multicomponent system involved in goal-directed behaviour that involves retaining and manipulating information (Baddeley, Hitch, & Allen, 2018; Chai, Abd Hamid, & Abdullah, 2018). A prominent model is that of (Baddeley and Hitch, 1974, Baddeley, 2000). The model describes four subcomponents: the phonological loop (based on vocalisation and rehearsal), visuospatial sketchpad (for visuo-spatial rehearsal), the central executive, and the episodic buffer (Baddeley, 2012). The limited capacities of the phonological loop and the visuospatial sketchpad are measured by tasks that require passive maintenance of verbal and visuo-spatial information respectively (often referred to as short-term memory). Frequently used tasks to assess short-term memory capacity are forward span tasks. The involvement of the central executive, which can be described as the attentional control system, is measured by working memory tasks that involve both maintenance and processing, for example backward span tasks. The episodic buffer is responsible for the binding of information in working memory, and linking working memory to perception and long-term memory (Baddeley, 2000). This buffer is difficult to assess by standardized and process-pure tasks (Nobre, Rodrigues, Sbicigo, Piccolo, Zortea, Junior, & de Salles, 2013) and its exact nature and properties have also been under debate (Heil, Rösler, & Rolke,  2003).

One of the reasons why there is no clear understanding of post-stroke working memory deficits is the heterogeneity in patient populations studied with respect to lesion locations, timing of assessment and small sample sizes. A second reason is that most studies only assess one component of working memory. A recent study (Karimian, Asgari, Neshat Doost, Oreizi, & Najafi, 2018) that investigated short-term memory, working memory, and long-term memory in the verbal, visuo-spatial, and visual domains in 35 stroke patients reports memory impairment on all aspects assessed. Most pronounced were impairments in visual short-term and long-term memory. A study by Nys and colleagues (2007) including 168 stroke patients reported a slightly higher percentage of patients with verbal memory impairment compared to visual memory impairment (25.6% and 22% respectively). In turn, a study in 39 acute stroke patients (Roussel, Dujardin, Hénon, & Godefroy, 2012) that investigated whether impairment in working memory remains after controlling for short-term memory capacity indicated that working memory impairment is a consequence of reduced short-term memory capacity.

A potential moderator of post-stroke working memory performance might be the time that elapsed since stroke. There is no consensus in the literature on working memory function in the chronic stage of stroke. For example, Kant, van den Berg, van Zandvoort, Frijns, Kappelle, & Postma, (2014) reported differences between chronic stroke patients and controls on all three working memory tasks they included, while McDonnell et al. (2011) did not find any differences between patients and controls. Yet another study (Andrade, Brucki, Bueno, & Siqueira Neto, 2012) reported only decreased working memory performance in patients who were classified as having post-stroke vascular dementia.

Although there is clear evidence that working memory is affected at least in the acute stage of stroke, a detailed analysis is lacking as to whether these deficits concern the different components and processing modes of the working memory system to a similar extent. Unravelling this in stroke patients is crucial as subsystems of working memory are essential for many other cognitive processes and may be closely related to functional outcome. The primary objective of this meta-analysis and systematic review is to quantify the severity of post-stroke working memory impairment by comparing patients to stroke-free controls. Specifically disentangling the effects for low-load working memory tasks (mainly addressing the passive limited capacity store) and more cognitively demanding high-load working memory tasks (involving executive processing). We will also systematically compare outcomes in the verbal and non-verbal domains, and in the sub-acute and chronic stages of stroke. A secondary objective is to identify possible associations between post-stroke working memory impairment and lesion location. As patient studies often have small samples and most studies do not include different aspects of working memory, quantitatively reviewing all available studies on this topic will help to provide a more comprehensive picture of working memory deficits after stroke.

## Method

PRISMA guidelines were used for the reporting of this systematic review (checklist provided in Appendix 1, Table 2; Moher, Liberati, Tetzlaff, & Altman, The PRISMA Group, 2009).

### Data Sources

Electronic database Pubmed was searched for relevant studies; last search was performed on 10–03-2019. The following search terms were used: “stroke”, “post-stroke”, “cva”, “cerebrovascular accident”, “cerebral vascular accident”, “brain infarct*”, “cerebral infarct*”, “brain lesion”, “ischemic lesion”, “cerebral ischemia”, “tia”, “transient ischemic attack” AND “memory disorders”, “memory”, “cognitive domain*”. Reference lists of selected articles were searched for potential missed articles.

### Study Selection and Eligibility Criteria

A two-step approach was used to select articles. Firstly, titles and abstracts of all search results were screened for the following characteristics by one reviewer (S.L.): (1) original article published in English, (2) participants are adults (> 18 years of age), (3) study concerning stroke or transient ischemic attack (TIA) patients, (4) sample size of at least 10 patients, as single-case studies or case series often concern rare cases whose behaviour might not be representative for the larger stroke patient population (5) outcome measures or descriptives include at least one working memory task; in case the abstract only mentioned memory function in general, the article was selected for full-text evaluation, (6) studies that only included patients with (subjective) memory complaints were excluded.

Secondly, 543 full-text articles were obtained from the selected studies and were reviewed on the following inclusion criteria: (7) a stroke-free control group was included as comparison, (8) clinical stroke patients in the sample (9) working memory function measured with a formal test or clearly described experimental paradigm, (10) treatment studies are included when baseline measures are reported. When two or more publications referring to the same sample were available, we extracted data only from the publication presenting the most accurate estimate, either because of sample size or outcome assessment. Two authors (S.L. and N.A.L.) independently performed the second step of the selection process. A meeting was held in case of disagreement which in all cases led to consensus.

Studies that included TIA patients were included in the meta-analysis as post-TIA cognitive impairment is often reported. A systematic review by Van Rooij, Kessels, Richard, De Leeuw, & van Dijk (2016) including 13 studies with data from 1,318 TIA patients, concluded that mild cognitive impairment is present in over a third of the TIA patients. When a mixed etiology lesion population was tested, the study was only included if separate results for the stroke patients could be retrieved.

### Data Extraction and Synthesis

Performance on working memory tests as compared to a healthy control group were extracted, as were participant characteristics, specific in- and exclusion criteria, and timing of assessment. First, overall performance on working memory tests was compared between stroke patients and healthy controls. Second, performances on low-load and high-load tasks were compared with a distinction between verbal and non-verbal tasks. Tests were considered low-load if they rely on remembering a limited amount of information over a short time, such as forward digit or spatial span tasks. In the working memory model of Baddeley and Hitch (1974) this is based on the phonological loop for verbal information and the visuo-spatial sketchpad for non-verbal information. Tasks that were considered high-load required some form of manipulation or updating, such as backward span or sequencing tasks, based on the central executive of the model. Tasks in which the stimuli (either visually or auditory) were digits, words, sentences or stories were categorized as verbal in nature. Tests were nonverbal in nature if stimuli were pictures of objects, scenes, line drawings or abstract figures. Third, a sub-analysis was conducted to examine the effect of timing of assessment (i.e. duration post stroke). Assessment within the first three months after stroke was considered sub-acute. Assessment after three months was considered as chronic. Qualitative synthesis of study results was performed with attention to lesion location. Working memory performance was compared between studies with specific inclusion criteria based on lesion location or imaging analyses relating lesion location to working memory performance. Working memory performance was compared within studies that selected groups based on lesion location.

### Risk of Bias Assessment

Risk of bias assessment was performed with the Research Triangle Institute (RTI) item bank, a tool to evaluate the bias and quality of observational studies (Viswanathan & Berkman, 2012; Viswanathan, Berkman, Dryden, & Hartling, 2013). As suggested by the RTI developers, slight adjustments were made to match the designs of the included studies (Appendix 2). Four items were dropped and items were reformulated to fit a case–control design. Ten items that assessed the selection bias, detection bias, attrition bias, selective outcome reporting and confounding were selected.

### Statistical Analysis

For the meta-analysis, sample sizes, means and standard deviations from the working memory tests were extracted from the studies. If necessary, corresponding authors were contacted to obtain these statistics based on the raw data. Summary statistics (Hedges’ *g*) were calculated based on sample sizes (*N*), means of patients and controls (*M*) and standard deviations (*SD*) with the following formula: *g* = StdDiff × *J*. StdDiff was calculated using the following formula: (*M*1—*M*2) / *SD*_pooled_, with *SD*_pooled_ = √(((*N*1-1) × *SD*1^2 + (*N*2 -1) × *SD*2^2) / (*N*1 + *N*2 -2)). The correction factor for different sample sizes is *J*, that was calculated as: 1—(3 / (4 × df—1)), where df = *N*1 + *N*2—2. Pooled variance was calculated by: √(1 / *N*1 + 1 / *N*2) × *SD*_pooled_. In case more than one outcome measure was reported, the average ES was calculated for the overall analysis. By using the mean of different outcome measures to calculate the effect size of the study we assume a correlation of 1 between different measures. This is a conservative approach as the actual correlation is probably less than 1 and the variance is lower than what we assume. The alternative is treating every outcome measure as fully independent, assuming a correlation of 0, which results in under-estimation of variance of the summary effect size. The actual overall effect size might therefore be slightly higher than our estimate. For the mixed-effect analyses of the effect of load and modality it was necessary to assume independence of outcome measures. Effect sizes were interpreted according to Cohen’s (1992) convention of small (0.20), medium (0.50), and large (0.80) effects for positive and negative values. Performance of stroke patients is lower compared to controls if the effect size (ES) is negative. Bias due to small sample sizes was corrected for by including sample size as a weighting factor (Hedges & Olkin, 1985). Random-effects models were used because of heterogeneity in populations studied and in outcome measures. Additionally, the goal is to generalize the results beyond the observed studies (Borenstein, Hedges, Higgins, & Rothstein, 2011). Heterogeneity was checked for by the use of the chi-square homogeneity test (Q). The fail-safe *N* was calculated for each study and a funnel plot was made (Rosenthal, 1991). The fail-safe *N* must to be larger than (5 × *k*) + 10, where *k* refers to the number of studies included in the meta-analysis (Clark-Carter, 2010). This measure gives an indication of how many studies with null-results should be unpublished due to publication bias to nullify the effect. All analyses were performed using Comprehensive Meta-Analysis version 2.0 (Engelwood, NJ, USA, 2005).

## Results

### Selection of Articles

**Fig. 1:**
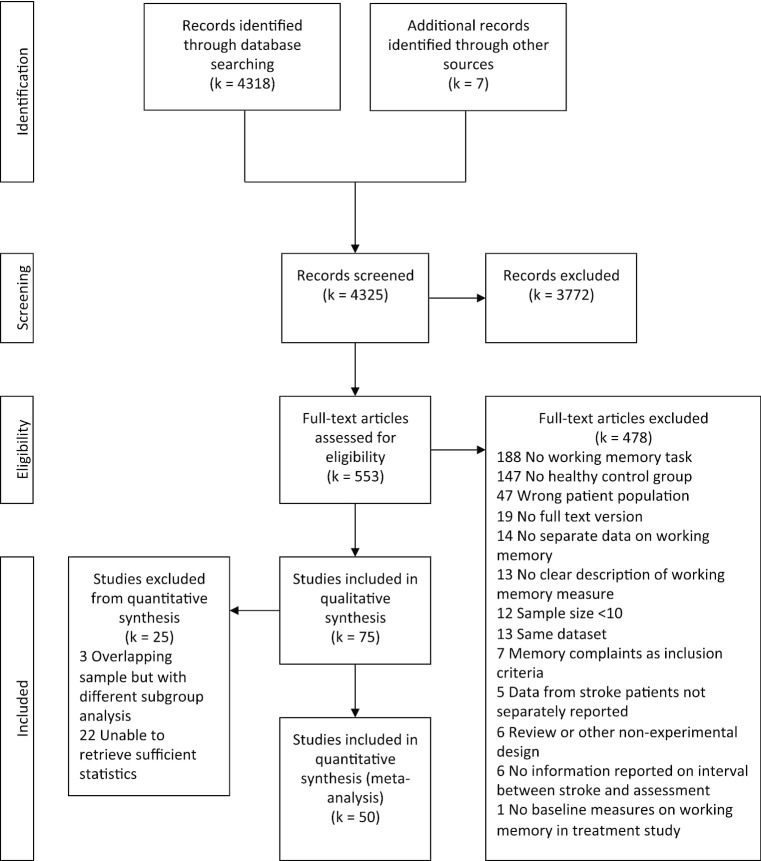
PRISMA Flowchart

The literature search resulted in 4,318 articles after removal of duplicates. Seven additional studies where identified by manually checking of reference lists of selected papers. In total, 553 were selected for full text screening. Seventy-five articles published between 1992 and early 2019 were eligible for inclusion. The eventual meta-analysis includes data of 3,084 stroke patients from 50 studies. An additional 25 studies of which the necessary statistics could not be retrieved or studies with overlapping samples but with relevant subgroup analyses were included in the systematic review. Figure 1 shows the flowchart of the literature search. Table S1 (online supplemental materials) shows the details of the studies included, these are: sample size and specific inclusion criteria, stroke type, age, interval between stroke and assessment, inclusion of prior stroke patients, inclusion of patients with pre-existing dementia, task load, task, Hedges *g*, and variance, and number of effect sizes for each primary study.

### Description of Study Populations

Ischemic stroke was the inclusion criterion for 30.6% (*k* = 23) of the studies. A quarter of the studies (24.0%, *k* = 18) included both patients with haemorrhagic and ischemic stroke. Concerning studies that included TIA patients and minor stroke, 5.3% (*k* = 4) included both stroke and TIA patients, 2.7% (*k* = 2) reported on patients with minor stroke,^1^ and 2.7% (*k* = 2) included only patients with transient ischemic attack (TIA). One study (1.3%, *k* = 1) included only patients with haemorrhagic stroke. A third (33.3%, *k* = 25) did not specify stroke type. A majority of the studies (61.3%, *k* = 46) did not select patients based on stroke location. Some studies (14.7%, *k* = 11) included only patients with subcortical lesions. Only one community-based study was fulfilled the inclusion criteria, all other studies were hospital- or rehabilitation center-based. Twenty percent (*k* = 10) studies included in the meta-analysis reported only one working memory measure. The mean number of outcome measures per study was 3.56. The maximum F number of outcome measures per study was 14. Comparisons between left and right hemisphere stroke were made in 16% of the studies (*k* = 12). Few studies included patients with stroke in one specific hemisphere (left: 6.7%,* k* = 5, right: 9.3%, *k* = 7). A total of 50 authors were approached to obtain additional information and necessary statistics from tasks separately (42.0%, *k* = 21), from stroke patients separately (12.0%, *k* = 6), means and standard deviations that were not reported (32%, *k* = 16), clarification on tasks or population (14%, *k* = 7). This resulted in 16 studies that could be included in the meta-analysis. Of the 34 articles of which the authors did not respond, 22 were included in the qualitative analyses.

### Risk of Bias Assessment

**Fig. 2:**
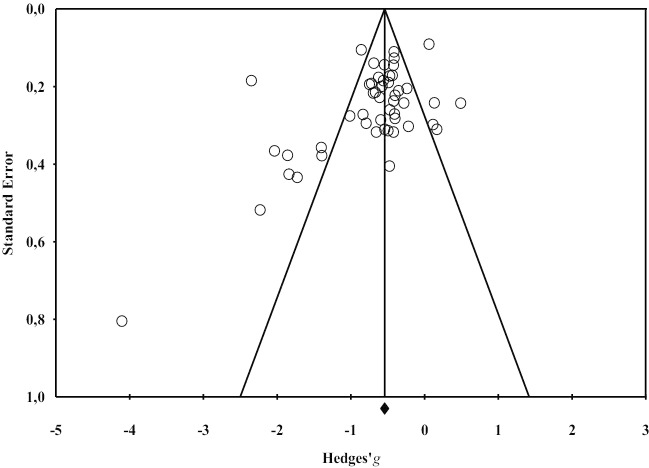
Funnel plot

Risk of bias assessment of individual studies based on the RTI items (see Appendix 2 and online supplemental materials Table S2) showed an unclear or high risk of confounding, as in more than two-third (72.0%, *k* = 54) of the studies details on possible prior strokes or pre-existing dementia were not specified, and in almost half (56.7%, *k* = 35) of the studies, in- and exclusion criteria for healthy controls were unclear. Although not invalidating the results of individual studies, it increased between-study heterogeneity. A majority of the studies (72.0%, *k* = 54) did include an age and education matched healthy control group. A second source of heterogeneity is the large variability in the intervals between stroke and assessment, both between and within studies. Almost all studies bear the risk of confirmation bias; only 5% of the studies (*k* = 4) reported that assessors were blinded to the status of the participant (patient or control). As deficits are often prominent, it is unsurprising that other studies did not report assessors to be naive to the participants group. The funnel plot shows the relation between the sample size and the effect size (Fig. 2). The plot shows some asymmetry, which may partly be due to heterogeneity in outcome measures and partly be due to publication bias. Especially the lower right corner is empty, which indicates that there are no small studies with small effect sizes. The summary statistics of the meta-analysis as shown in Table 1 includes the *Fail-safe N* for each analysis as the number of studies with an effect size of zero that should be added to lose the significant result. All analyses have a *Fail-safe N* larger than the pre-set criterion, indicating that it is unlikely that the effect is only significant because of publication bias.

**Table 1:** Results of the meta-analyses

	*k*	*N P/HC*	*ES (g)*	*95% CI*	*Q*	*p (Q)*	*I*^*2*^	τ^*2*^	*Fail-safe N*
Overall	50	3,084/ 2,898	-0.65	-0.80 to -0.51	287.86	< 0.001	82.98	0.21	4,949
Low- load	39	2,699/ 2,318	-0.58	-0.77 to -0.40	308.16	< 0.001	87.67	0.28	2,411
High- load	41	2,475/ 2,308	-0.59	-0.73 to -0.45	171.72	< 0.001	76.71	0.14	2,783
Sub-acute	14	830/ 466	-0.43	-0.68 to -0.19	45.80	< 0.001	71.62	0.15	144
Chronic	22	1,165/ 1,377	-0.90	-1.15 to -0.65	157.25	< 0.001	86.65	0.29	1,740

*Note. k* = number of studies; P = patients; HC = healthy controls.

### Overall Effect, Effect of Memory Load, Modality and Timing of Assessment

**Fig. 3:**
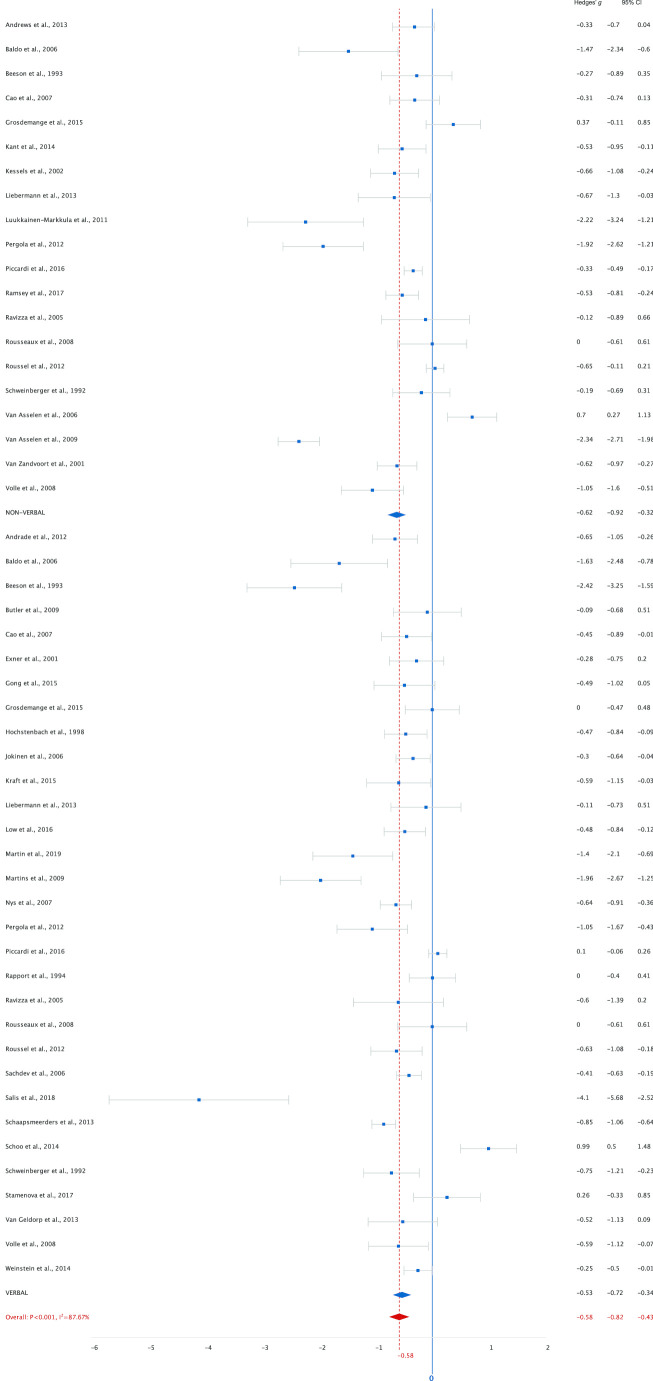
Performance on low-load tasks categorized by modality (verbal and non-verbal)

**Fig. 4:**
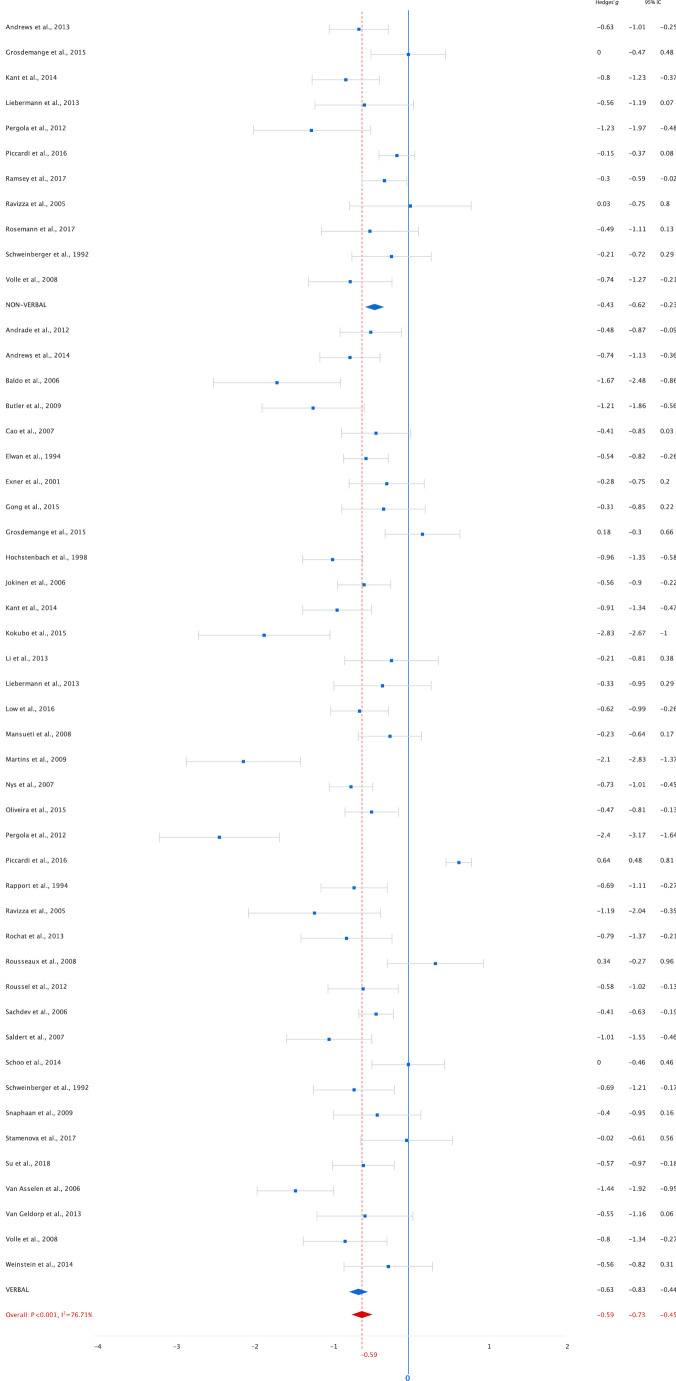
Performance on high-load tasks categorized by modality (verbal and non-verbal)

**Fig. 5:**
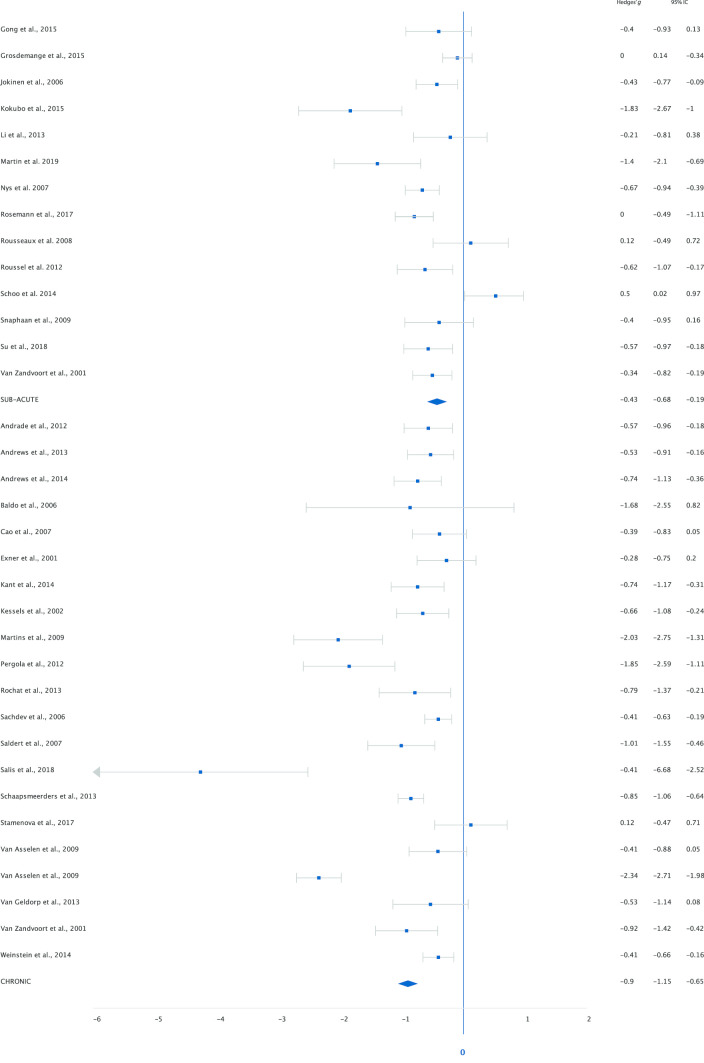
Overall working memory performance categorized by interval between stroke and assessment (sub-acute < 3 months and chronic)

Analysis of data from 50 studies including 3,084 patients and 2,898 healthy controls on working memory averaged across tasks resulted in an overall moderate ES of -0.65 ([− 0.80 to − 0.51], *p* < 0.001) for lower working memory performance in stroke patients. Thirty-nine studies (78%) included a low-load working memory task. The analysis showed a moderate ES of -0.58 ([-0.77 to -0.40], *p* < 0.001). Analysis of forty-one studies (82%) with a high-load working memory task resulted in a comparable ES of -0.59 ([-0.73 to -0.45]), *p* < 0.001). Figures 3 and 4 show the effect sizes per study grouped by modality under high and low-load conditions separately. Analyses show medium effect sizes (< -0.50) in the verbal domain for high-load (-0.63) and low-load (-0.53) tasks and for low-load (-0.62) tasks in the non-verbal domain. For high-load non-verbal tasks the effect size was slightly lower (-0.43). In order to compare the effect of low- and high-load we needed to assume independence of outcome measures. A mixed-effects meta-analysis shows a *Q* statistic for the difference between the effect-sizes of 0.009 (*p* = 0.922), indicating that there were neither differences in effect sizes between low- and high-load tasks, nor differences in effect sizes for non-verbal and verbal tasks (high-load *Q* = 2.16, *p* = 0.142; low-load *Q* = 0.24, *p* = 0.626). To examine the effect of interval between stroke onset and assessment, a secondary analysis was performed. For this analysis we excluded studies that analysed patients in the sub-acute and chronic stage as one group. Fourteen studies (28%) included patients in the sub-acute stage. The analysis showed a moderate ES of -0.43 ([-0.68 to -0.19], *p* < 0.001). For patients in the chronic stage (*k* = 22, 44%) the ES was large, -0.90 ([-1.15 to -0.65], *p* < 0.001, Fig. 5). A mixed-effects analysis showed that this difference in effect-sizes is statistically significant, *Q* = 6.88, *p* = 0.009. Patients in the chronic stage show more decrement in working memory performance compared to healthy controls than patients in the sub-acute stage. The heterogeneity indices (*Q*) were all statistically significant (*p* < 0.05) for all analyses, indicating variation in study outcomes. The between study variance (τ ^2^) of all studies is estimated as 0.21. The I^2^ of 82.98 indicates that most of the observed variance reflects differences in study effect rather than sampling error. Results of the overall analysis and sub-analyses are presented in Table 1. Exclusion of studies that included patients with TIA did not lead to different results (Table S3a online supplemental materials). To minimize heterogeneity due to task variation, we reran the analysis including only studies with Digit Span or Spatial Span as working memory measure. Effect sizes were highly similar to those of the analysis including all studies for the overall, and low- and high-load analyses. The sub-analysis with patients in the sub-acute stage included only eight studies and yielded a lower effect size (Table S3b online supplemental materials).

### Qualitative Assessment of Studies Comparing Lesion Location and Longitudinal Studies

This qualitative assessment includes 75 studies, 50 from the meta-analysis and 25 additional studies that were not taken into account in the meta-analysis due to missing statistics or overlapping samples. Sixteen percent of the studies (*k* = 12) compared working memory performance of patients with a left hemisphere stroke to patients with a right hemisphere stroke. Two-third of them (66.7%, *k* = 8) did not report a statistically significant difference in performance between left and right hemisphere stroke patients. Twenty-five percent (*k* = 3) reported a worse performance in left hemisphere stroke patients compared to right hemisphere stroke patients and controls on immediate serial recall tasks (Ho, Kong, & Koon, 2018), on letter-number sequencing (Andrews, Halford, Shum, Maujean, Chappell, & Birney, 2014), and on a visually presented digit span forward and backward task (Low, Crewther, Perre, Ong, Laycock, & Wijeratne, 2016). One study (8.3%) reported no difference in performance of left hemisphere stroke patients and controls but impaired performance in right hemisphere stroke patients on a backward spatial span task (Van der Ham, van Wezel, Oleksiak, van Zandvoort, Frijns, Kappelle, & Postma, 2012).

A second comparison made in studies is between patients with an anterior and posterior lesion. Studies showed performance more strongly affected in patients with frontal lesions compared to posterior lesions on different forward span tasks (Roussel, Dujardin, Hénon, & Godefroy, 2012), on digit span backward (but not forward, Leskelä et al., 1999), and on high-load n-back tasks (Andrews, Halford, Shum, Maujean, Chappell, & Birney, 2013). In contrast, two studies reported the opposite; one reported performance in the posterior group to be inferior to patients with anterior lesions on digit span forward, with no differences on spatial span (Beeson, Bayles, Rubens, & Kaszniak, 1993). The other study reported lower performance in patients with inferior parietal lesions compared to inferior frontal lesions on several forward span tasks (Baldo & Dronkers, 2006).

**Fig. 6:**
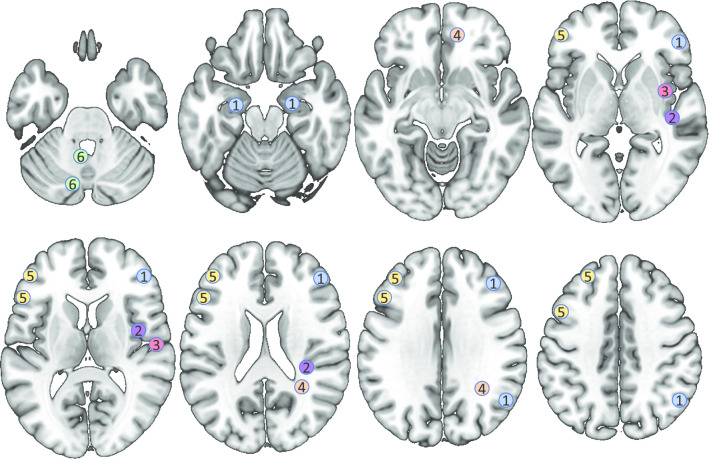
Neural correlates associated with working memory performance. 1. Van Asselen et al., 2006, anatomical description: posterior parietal cortex and dorsolateral prefrontal cortex in the right hemisphere, hippocampal formation bilateral. 2. Malhotra et al., 2005, MNI coordinates: x = 35, y = -30, z = 24; x = 44, y = -12, z = 16, x = 43, y = -19, z = 04. 3. Hirel et al., 2017, MNI coordinates: x = 59, y = -12, z = 11; x = 39, y = -2, z = -6. 4. Roussel et al., 2012, anatomical description: frontobasal and posterior centrum semi-ovale regions in the right hemisphere. 5. Volle et al., 2008, Brodmann areas: 6, 8, 9, 44, 45, 46. 6. Baier et al., 2014, MNI coordinates: x = − 6, y = − 53, z = − 39; x = − 8, y = − 73, z = − 42; x = − 9, y = − 71, z = − 42; x = − 5, y = − 67, z = − 42.

Whereas 59 studies used neuroimaging to confirm stroke, to check for exclusion criteria and to describe the sample or to create subgroups, only seven studies related specific lesion locations to working memory performance. Spatial working memory performance was associated with lesions in the right posterior parietal and right dorsolateral prefrontal cortex and bilaterally in the hippocampal formation (Van Asselen, Kessels, Neggers, Kappelle, Frijns, & Postma, 2006). Both parietal white matter and insula lesions were associated with spatial working memory deficits in neglect patients (Malhotra, Jäger, Parton, Greenwood, Playford, Brown, & Husain, 2005). Not only spatial, but also verbal short-term memory was associated with parietal lesions. Lesions in the insula were in the same study associated with a lower performance in a musical working memory task (Hirel, Nighoghossian, Lévêque, Hannoun, Fornoni, Daligault, & Caclin, 2017). Another study demonstrated that high-load working memory tasks were associated with lesions in both the frontobasal and posterior centrum semi-ovale regions (Roussel et al., 2012). A study that only included patients with frontal lesions reported that the posterior part of the left middle frontal gyrus is significant for high-load but not for low-load working memory tasks (Volle, Kinkingnéhun, Pochon, Mondon, Thiebaut de Schotten, Seassau, & Levy, 2008). A study with patients with cerebellar lesions attributed filtering of information in working memory tasks, but not working memory capacity, to specific areas of the cerebellum, such as the tonsil, the inferior semilunar lobule, and parts of the vermal pyramid (Baier, Müller, & Dieterich, 2014). Finally, one study reported no predictive effect of lesion topography on memory. However, this study used a combined measure of spatial and verbal recall, recognition and working memory (Ramsey, Siegel, Lang, Strube, Shulman, & Corbetta, 2017). Figure 6 shows how the results of these neuroimaging studies relate to each other.

Concerning timing of post-stroke working memory assessment, only four studies employed a longitudinal design. Three of these did not find any difference in performance of patients between the different time points. Two of these studies assessed the patients in the first week after stroke, with a follow up at three and six months respectively (Su, Guo, Zhang, Zhou, Chen, Zhou, & He, 2018; Van Zandvoort, De Haan, & Kappelle, 2001a, b). The third study performed the first assessment between three and six months, with a three-year follow-up (Sachdev, Chen, Brodaty, Thompson, Altendorf, & Wen, 2009). The fourth study reported improved performance measured over three intervals; at two weeks, three months, and 12 months (Ramsey et al., 2017).

## Discussion

This comprehensive review investigated working memory function post-stroke in comparison to healthy controls. A narrative and quantitative meta-analytic approach were combined. This allowed us to include studies of which we could not retrieve the necessary statistics for a meta-analysis but that did compare patients and controls on working memory performance. A meta-analytic approach was used for quantification of severity of working memory deficits after stroke. In addition, it allowed for a comparison of the effect of high- and low-load conditions, and of verbal and non-verbal tasks, in the sub-acute and chronic stages. The literature was systematically reviewed to gain insight in the effect of lesion location on working memory and findings from longitudinal studies. Of the 75 studies incorporated in this review, 50 studies were included in the meta-analysis. The meta-analysis revealed a moderate overall effect size, indicating lower working memory performance in post-stroke patients relative to stroke-free controls.

Categorical analyses showed that performance on low-load working memory tasks was impaired to the same extent as the performance on high-load tasks. This is in line with the study by Roussel and colleagues (2012) who concluded in their study that working memory deficits are a consequence of reduced short-term memory capacity. Their conclusion is based on the finding that when controlling for verbal memory span, the impairment on working memory span disappeared. In terms of the model by Baddeley and Hitch (1974), this indicates impairment in stroke patients in the phonological loop and visuospatial sketchpad rather than the central executive. However, as most of the high load tasks are a form of backward span tasks, it can be argued that the tax on the central executive is low as the serial order stays the same and only needs to be reversed. Especially for spatial span tasks, it has been suggested that this process may only rely on the visuo-spatial sketchpad, as the visual pattern is not affected by reversing (e.g. Kessels, van Den Berg, Ruis, & Brands, 2008; Wilde, Strauss, & Tulsky, 2004). The few studies in this review that included tasks with higher demands (n-back tasks and letter-number sequencing) show inconsistent results Three out of seven studies had considerable larger effect sizes and four had effect sizes comparable to the mean. Based on the current limited evidence, we have no strong reason to assume an additive deficit in more complex processing. However, the question of whether stroke results in an additive deficit in the central executive under more demanding conditions needs further investigation.

Stroke seems to affect both slave systems, the phonological loop and the visuo-spatial sketchpad, to the same extent. Effect sizes were comparable for verbal and non-verbal tasks. Out of 12 studies that compared patients with left and right hemispheric stroke, eight did not report differences in performance based on hemisphere of the lesion. This is not in line with the theory of hemispheric specialization, which predicts more severe impairments in verbal working memory in patients with a left hemisphere stroke and more spatial working memory impairments in patients with right hemisphere stroke (e.g. Habib, Nyberg, & Tulving, 2003). However, studies that did report a difference were in concordance with the theory of hemispheric specialization; one reported inferior performance in right hemisphere lesioned patients on a spatial task, three reported inferior performance on verbal tasks in patients with left hemisphere stroke. Based on the studies reviewed here we conclude that both frontal and non-frontal lesions, especially posterior parietal lesions, affect working memory performance. However, as with effect of hemisphere, there is no consensus on specialized areas depending on working memory task characteristics. A recent study indicates that whereas visual and motor deficits can be well explained by lesion characteristics, visual and verbal memory deficits are better predicted by measures of functional connectivity (Siegel, Ramsey, Snyder, Metcalf, Chacko, Weinberger, & Corbetta, 2016). Our results support the view of a bilateral fronto-parietal network involved in working memory. Involvement of a widespread network might explain the high frequency of working memory deficits after stroke and the global nature of working memory impairment that we show in our meta-analysis.

Concerning the possible moderating effect of time between stroke and assessment, the meta-analysis and systematic review resulted in different conclusions. The meta-analysis showed a larger effect size in patients in the chronic stage of stroke compared to the sub-acute stage. This can be interpreted in different ways. First, remote effects may increase over time. A recent longitudinal study with MRI scans at one month, three months and twelve months after stroke, showed secondary degeneration in the limbic system and increased mean diffusivity after cortical stroke, independent of lesion location. The clinical outcome measure was the National Institutes of Health Stroke Scale (NIHSS) score, which did improve during the follow-up (Haque, Gabr, Hasan, George, Arevalo, Zha, & Satani, 2019). No cognitive measures were taken in this study. Lower remote white matter integrity was related to worse long-term cognitive performance in a study with a follow-up 11 years after stroke (Schaapsmeerders, Tuladhar, Arntz, Franssen, Maaijwee, Rutten Jacobs, & de Leeuw, 2016). Second, selection bias may play a role. In the acute stage, patients with more extensive lesions or severe aphasia are less likely to be included in research, whereas they may be able to participate in research months later. This might lead to underestimation of working memory deficits in the acute stage. In addition, patients with good recovery might not participate months after the event, because they resumed their daily activities. Of the four longitudinal studies included in our review, three showed impaired performance in working memory that remained stable up to three years after the event. One longitudinal study showed spontaneous recovery with performance improving over the course of one year.

To pull apart these different explanations and gain more insight in the time-course of working memory deficits after stroke, more longitudinal research is needed. A limitation of the studies in the current meta-analysis is that they did not allow for a more fine-grained analysis of the effect of post-stroke interval. As the range of intervals within studies was very high, a categorical comparison was more informative. A second study-related limitation is that many studies did not specify whether they included patients with pre-existing cognitive decline that could have influenced working memory performance. With respect to the current review, a limitation is that only Pubmed was used for the systematic search. Future studies should focus more on structural and functional connectivity in relation to working memory performance after stroke. These techniques could help to identify who is at risk for little spontaneous recovery or even deterioration in working memory and thereby guide rehabilitation programmes.

## Conclusion

Taken together, this meta-analysis and systematic review clearly demonstrate the global nature of the impairment in working memory post-stroke. All subsystems of working memory are affected evidently and similar findings were reported for non-verbal and verbal tasks. Lesions in a widespread fronto-parietal network result in working memory impairment, which in turn results in a reduced capability to maintain verbal and non-verbal information. The finding that effect sizes are larger in the chronic stage compared to the sub-acute stage and that most longitudinal studies show no improvement in working memory performance, is important to take into account when discussing future prognosis with patients.

### Electronic supplementary material

Below is the link to the electronic supplementary material.

Supplementary file1 (DOCX 41.7 kb)

## Appendix 1

See Table 2.

**Table 2:** PRISMA Checklist

Section/topic	#	Checklist item	Reported on page #
**TITLE**			
Title	1	Identify the report as a systematic review, meta-analysis, or both	Title page
**ABSTRACT**			
Structured summary	2	Provide a structured summary including, as applicable: background; objectives; data sources; study eligibility criteria, participants, and interventions; study appraisal and synthesis methods; results; limitations; conclusions and implications of key findings; systematic review registration number	1
**INTRODUCTION**			
Rationale	3	Describe the rationale for the review in the context of what is already known	2–4
Objectives	4	Provide an explicit statement of questions being addressed with reference to participants, interventions, comparisons, outcomes, and study design (PICOS)	4
**METHODS**			
Protocol and registration	5	Indicate if a review protocol exists, if and where it can be accessed (e.g., Web address), and, if available, provide registration information including registration number	N/A
Eligibility criteria	6	Specify study characteristics (e.g., PICOS, length of follow-up) and report characteristics (e.g., years considered, language, publication status) used as criteria for eligibility, giving rationale	5, 6
Information sources	7	Describe all information sources (e.g., databases with dates of coverage, contact with study authors to identify additional studies) in the search and date last searched	5–8
Search	8	Present full electronic search strategy for at least one database, including any limits used, such that it could be repeated	5
Study selection	9	State the process for selecting studies (i.e., screening, eligibility, included in systematic review, and, if applicable, included in the meta-analysis)	Figure 1
Data collection process	10	Describe method of data extraction from reports (e.g., piloted forms, independently, in duplicate) and any processes for obtaining and confirming data from investigators	5–8
Data items	11	List and define all variables for which data were sought (e.g., PICOS, funding sources) and any assumptions and simplifications made	6–7
Risk of bias in individual studies	12	Describe methods used for assessing risk of bias of individual studies (including specification of whether this was done at the study or outcome level), and how this information is to be used in any data synthesis	7 and Appendix 2
Summary measures	13	State the principal summary measures (e.g., risk ratio, difference in means)	7, 8
Synthesis of results	14	Describe the methods of handling data and combining results of studies, if done, including measures of consistency (e.g., I^2^) for each meta-analysis	8
Risk of bias across studies	15	Specify any assessment of risk of bias that may affect the cumulative evidence (e.g., publication bias, selective reporting within studies)	Table S2 supplemental materials
Additional analyses	16	Describe methods of additional analyses (e.g., sensitivity or subgroup analyses, meta-regression), if done, indicating which were pre-specified	8
**RESULTS**			
Study selection	17	Give numbers of studies screened, assessed for eligibility, and included in the review, with reasons for exclusions at each stage, ideally with a flow diagram	9, Fig. 1
Study characteristics	18	For each study, present characteristics for which data were extracted (e.g., study size, PICOS, follow-up period) and provide the citations	9–10, Table S1 supplemental materials
Risk of bias within studies	19	Present data on risk of bias of each study and, if available, any outcome level assessment (see item 12)	11 and Table S2 supplemental materials
Results of individual studies	20	For all outcomes considered (benefits or harms), present, for each study: (a) simple summary data for each intervention group (b) effect estimates and confidence intervals, ideally with a forest plot	12–13, Table 1, Fig. 3–5
Synthesis of results	21	Present results of each meta-analysis done, including confidence intervals and measures of consistency	Table 1
Risk of bias across studies	22	Present results of any assessment of risk of bias across studies (see Item 15)	11 and Fig. 2
Additional analysis	23	Give results of additional analyses, if done (e.g., sensitivity or subgroup analyses, meta-regression [see Item 16])	12, 13, Fig. 3–5, Table S3a,b supplemental materials
**DISCUSSION**			
Summary of evidence	24	Summarize the main findings including the strength of evidence for each main outcome; consider their relevance to key groups (e.g., healthcare providers, users, and policy makers)	16–19
Limitations	25	Discuss limitations at study and outcome level (e.g., risk of bias), and at review-level (e.g., incomplete retrieval of identified research, reporting bias)	18, 19
Conclusions	26	Provide a general interpretation of the results in the context of other evidence, and implications for future research	16–19
**FUNDING**			
Funding	27	Describe sources of funding for the systematic review and other support (e.g., supply of data); role of funders for the systematic review	Title page and 19

## Appendix 2

### Adapted RTI Item Bank for Assessing Risk of Bias and Confounding

Q1. Are inclusion and exclusion criteria applied uniformly?

Consider patients vs. controls if applicable, otherwise individual patients

Q2. Is the recruitment strategy the same across individuals or study groups?

Consider patients vs. controls if applicable, otherwise individual patients

Q3. Is the selection of the comparison group adequate?

Age and education matched

Q5. Is the outcome assessor blinded to exposure status?

Assessor of cognitive function blinded for clinical status (stroke or not)?

Q6. Are valid and reliable measures implemented?

Reliable and conventional ascertainment of stroke?

Q7. Is the length of follow-up the same across individuals or study groups?

Delay from stroke to cognitive testing uniform for all individuals?

Q8. Is the impact of loss to follow-up assessed?

Only applicable if follow-up study

Q9. Are important primary outcomes reported?

Cognitive impairment

Timing of cognitive testing

Q13. Are important confounding variables taken into account in the design and analysis?

*Stratified by level of importance:*

1. *Age, level of education, prior stroke, pre-existing dementia.*
2. *Vascular risk factors, vascular brain damage, concurrent neuropsychiatric disturbances.*

Q4, Q10, Q11, Q12 of the RTI item bank are not relevant to the included studies

## Footnotes

1 Li, You, Xu, Yuan, Shen, Huang, & Liu (2019) defined minor stroke by a score of 5 or less on the National Institute of Health Stroke Scale at admission and a diagnosis of ischemic stroke confirmed by neuroimaging; Mansueti, De Frias, Bub, and Dixon (2008) based inclusion on self-reported mild stroke for which medical attention was received, in a community-based study.
