# Supplementary material for: Post-Stroke Working Memory Dysfunction: A Meta-Analysis and Systematic Review
Source: Neuropsychol Rev. 2020 Nov 24;31(1):202–19. doi: 10.1007/s11065-020-09462-4 (PMC7889582; doi:10.1007/s11065-020-09462-4)
Supplement: Supplementary file 1 — Supplementary file1 (DOCX 41.7 kb) [file 11065_2020_9462_MOESM1_ESM.docx]

**Online Supplemental Materials**

**Table S1**

*Overview of articles included.*

| **Study** | **Patients *N***  **subgroups/ specific incl.** | **Stroke type** | **Age *M* (*SD*) or Median [range]** | **Interval^1^ in months  *M* (*SD*) [range]** | **Prior stroke** | **Prior dementia** | **Healthy controls** | **Low-/ high-load** | **Task** | **Hedges’s *g*, Variance** | **Number**  **of ES** |  |  |
| --- | --- | --- | --- | --- | --- | --- | --- | --- | --- | --- | --- | --- | --- |
| Andrade et al., 2012* | 50 subcortical | IS | 66.0 (8.9) | > 3 | NS | NS | 50 | both | DSF, DSB | -.57  .04 | 6 |  |  |
| Andrews et al., 2013* | 14 frontal, 30 non-frontal | NS | 64.4 (12.0) | 88 (60) | NS | NS | 41 | both | 1-, 2-, 3-back | -.48  .04 | 6 |  |  |
| Andrews et al., 2014* | 21 LH, 20 RH | NS | 65.1 (12.1) | 87 (61) | NS | NS | 41 | high | Letter-Number Sequencing | -.74  .04 | 2 |  |  |
| Baier et al., 2014 | 8 LH, 21 RH | IS | Median 56 [20-81] | < .5 | NS | NS | 10 | low | Experimental visuospatial task | NA | NA |  |  |
| Baldo et al., 2006* | 10 left IPC, 8 left IFC | NS | 61.1 (8.0) | 48 (44) | no | no | 6 | both | SSF, word span, DSF, digit span with a pointing response, 2-back task | -1.72  .19 | 10 |  |  |
| Beeson et al., 1993* | 7 anterior LH, 7 posterior LH | NS | 65.3 (7.0) | 44 (NS) | no | no | 14 | low | DSF, SSF | -1.39  .14 | 4 |  |  |
| Bugarski Ignjatovic et al., 2015 | 40 | IS | Median NS [45-78] | < 1 | no | no | 40 | low | DSF, SSF | NA | NA |  |  |
| Burton et al., 2004 | 69 | NS | 80.2 (4.1) | 3 (NS) | NS | no | 23 | low | memory scanning (CDR) | NA | NA |  |  |
| Butler et al., 2009* | 9 neglect, 11 RH | NS | 61.1 (14.9) | 2.6 (1.7) | NS | NS | 10 | both | DSF, DSB | -.65  .10 | 4 |  |  |
| Cao et al., 2007* | 40 | IS | 38.8 (8.3) | 9.2 [6-12] | no | NS | 40 | both | DSF, DSB, SSF | -.40  .05 | 3 |  |  |
| Danet et al., 2015 | 12 left thalamic | IS | 53.2 (14.6) | 19 [3-59] | no | no | 25 | both | DSF, SDB, SSF, SSB | NA | NA |  |  |
| Elwan et al., 1994* | 23 acute, 34 chronic | IS and TIA | 59.1 (7.9) | acute < 1; chronic 12 (12) | NS | no | 30 | high | PASAT | -.54  .02 | 3 |  |  |
| Exner et al., 2001* | 15 thalamic, 22 basal ganglia | IS and HS | 54.8 (10.5) | 36 (34) | no | no | 15 | both | DSF, DSB | -.28  .06 | 4 |  |  |
| Fu et al., 2017 | 34 brainstem | NS | 59.6 (12.7) | < 1 | no | no | 26 | both | DST | NA | NA |  |  |
| Godefroy et al., 1994 | 11 lenticulostriate | NS | 56.5 (NS) | [1.5 – 2] | no | no | 11 | low | DSF, SSF | NA | NA |  |  |
| Gong et al., 2015* | 25 occipital | NS | 60.9 (11.0) | [.5 – 2] | NS | no | 29 | both | DSF, DSB | -.40  .07 | 2 |  |  |
| Gorišek et al., 2016 | 10 left MCA | IS | 67 (NS) | 1.8 (1) | no | no | 10 | low | Sternberg task | NA | NA |  |  |
| Grosdemange et al., 2015* | 28 | IS | 67.5 (8.3) | < 1 | no | no | 41 | both | DSF, DSB, SSF, SSB | .14  .06 | 4 |  |  |
| Hirel et al., 2017 | 10 LH, 10 RH | IS | 60.6 (11.7) | 17 (15) | no | no | 14 | low | experimental word, tone, visual STM tasks | NA | NA |  |  |
| Ho et al., 2018 | 15 LH, 10 RH | NS | 56.3 (8.9) | 92 (78) | no | NS | 25 | low | ISR 7 variants | NA | NA |  |  |
| Hochstenbach et al., 1998a* | 199 | IS and HS | 55.9 (11.2) | 2.4 (1.6) | yes | NS | 32 | both | DSF, DSB | -.72  .04 | 2 |  |  |
| Hochstenbach et al., 1998b | 12 basal ganglia | IS and HS | 53.4 (NS) | < 3 | NS | NS | 24 | both | DSF, DSB | NA | NA |  |  |
| Jokinen et al., 2006* | 321 | IS | 70.3 (7.6) | 3 (NS) | yes | yes | 38 | both | DSF, DSB | -.43  .03 | 2 |  |  |
| Kant et al., 2014* | 39 | IS and HS | 58.2 (14.2) | 17 (8.3) | NS | no | 53 | both | Letter Number Sequencing, SSF, SS | -.69  .05 | 3 |  |  |
| Karimian et al., 2018 | 35 | IS | 61.7 (NS) | < 12 | no | no | 35 | both | DSF, SDB, SSF, SSB | NA | NA |  |  |
| Kessels et al., 2002* | 28 LH, 16 RH, 6 BL | IS | 52.4 (13.0) | 26 (22) | no | no | 39 | low | SSF | -.66  .05 | 1 |  |  |
| Kober et al., 2015 | 24 | NS | Median NS [37-82] | > 1 | no | no | 40 | both | DSF, DSB, SSF, SSB | NA | NA |  |  |
| Kokubo et al., 2015* | 15 putaminal | HS | 53.0 (10.2) | 2 (.2) | no | no | 15 | high | Letter Number Sequencing | -1.84  .18 | 1 |  |  |
| Kraft et al., 2015* | 16 thalamic | NS | 41.2 (11.0) | 36 (42) | no | no | 52 | low | Experimental task based on TVA | -.59  .08 | 1 |  |  |
| Leskelä et al., 1999 | 62 frontal, 188 non-frontal | IS | 70.5 (7.6) | 3.4 (.4) | yes | yes | 39 | both | DSF, DSB | NA | NA |  |  |
| Li et al., 2013* | 21 | TIA | 50.1 (6.5) | < 1 | no | NS | 21 | high | DSB | -.21  .09 | 1 |  |  |
| Li et al., 2019 | 68 | minor stroke | 60.3 (12.1) | < .5 | no | no | 36 | both | DSF, DSB | NA | NA |  |  |
| Liebermann et al., 2013* | 19 thalamic | IS | 44.6 (11.1) | 29 (35) | NS | NS | 20 | both | DFS, DSB, SSF, SSB | -.42  .10 | 4 |  |  |
| Low et al., 2016* | 12 LH, 15 RH, 3 RH neglect, 10 TIA | IS and TIA | 58.3 (9.4) | 7.0 (3.9) IS; 3.9 (1.9) TIA; T2 + 3 | no | no | 31 | both | visual variant of DSF and DSB | -.55  .04 | 8 |  |  |
| Luukkainen-Markkula et al., 2011 | 11 RH neglect | NS | 58.7 (9.8) | 2.5 (2.1) | no | NS | 12 | low | SSF | -2.23  .27 | 1 |  |  |
| Malhotra et al., 2005 | 10 RH, 10 RH neglect | NS | 66.0 (13.9) | 67 (58) | NS | NS | 10 old 10 young | low | computerized spatial span task | NA | NA |  |  |
| Malm et al., 1998 | 24 cerebellum or brainstem | IS | 36.9 (6.5) | .5 (NS) | NS | NS | 14 | low | DSF, DSB, sentence span task, word span | NA | NA |  |  |
| Malouin et al., 2004 | 12 supratentorial | NS | 56.1 (9.9) | 18 (14) | no | NS | 14 | low | forward visuospatial, verbal, and kinesthetic span tasks | NA | NA |  |  |
| Mansueti et al., 2008* | 24 study 1, 24 study 2 | minor stroke | 71.0 (9.2) | Self-report | no | no | Study 1 24 Study 2 24 | high | reading span, computational span | -.23  .04 | 6 |  |  |
| Martin et al., 2019* | 32 LH | IS | 63.5 (12.7) | < .25 | no | no | 13 | low | digit match span, category probe span | -1.40  .13 | 2 |  |  |
| Martins et al., 2009* | 22 LH | NS | Median NS [31-80] | >3 | no | NS | 22 | both | DSF, DSB | -2.03  .14 | 2 |  |  |
| McDonnel et al., 2011 | 17 | IS and HS | 70.1 (NS) | 107 (NS) | NS | no | 13 | both | DSF, DSB, SSF, SSB, PASAT | NA | NA |  |  |
| Nys et al., 2007* | 168 | IS and HS | 62.7 (13.8) | < .75 | no | no | 77 | both | DST, SSF | -.68  .02 | 3 |  |  |
| Oliveira et al., 2015* | 42 RH | IS and HS | 58.3 (11.8) | 20 (24) | no | no | 84 | high | ADO, AS | -.47  .03 | 4 |  |  |
| Pergola et al., 2012* | 9 paramedian, 8 tuberothalamic | IS | 62 (12) | 67 (48) | no | no | 28 | both | DSF, SDB, SSF, SSB | -1.85  .14 | 8 |  |  |
| Piccardi et al., 2016* | 346 | NS | 63.7 (14.3) | 2.2 (7.2) | no | no | 272 | both | DSF, SDB, SSF, SSB | .07  .01 | 4 |  |  |
| Planton et al., 2012 | 60 | IS | 59.7 (14) | 3.6 (.7) | no | no | 40 | both | DSF, SDB, SSF, SSB | NA | NA |  |  |
| Pluta et al., 2017 | 29 RH, 24 LH, 5 BL | NS | 57.7 (12.9) | 25 (31) | no | no | 33 | both | DST | NA | NA |  |  |
| Ramsey et al., 2017* | T1 108, T2 91, T3 88 | IS and HS | 54 (10) | .5, 3, 12 | no | no | 31 | both | SSF, SSB | -.42  .02 | 4 |  |  |
| Rapport et al., 1994* | 29 RH neglect, 22 RH no neglect | NS | 62.2 (6.7) | 5.8 (10.1) | NS | NS | 20 | both | DSF, DSB | -.35  .05 | 4 |  |  |
| Ravizza et al., 2005* | 10 cerebellar | IS | 61.3 (16.6) | chronic, NS | NS | NS | 15 | both | DSF, DSB, SSF, SSB | -.47  .17 | 4 |  |  |
| Rochat et al., 2013* | 55 | IS and HS | 56.4 (13.1) | 24.1 (36.1) | no | no | 15 | high | Letter-Number Sequencing | -.79  .09 | 1 |  |  |
| Rosemann et al., 2017* | 20 MCA | NS | 52 (9.8) | < .25 | no | no | 20 | high | 2-back | -.49  .10 | 2 |  |  |
| Rousseaux et al., 2008* | 20 AChAI | NS | 59.6 (12.39) | 1.5 (.6) | no | no | 20 | both | DSF, SDB, SSF | .17  .10 | 3 |  |  |
| Roussel et al., 2012* | 17 frontal, 12 posterior | IS and HS | 45.5 (12.9) | < 1 | no | no | 29 | both | DSF, DSB, experimental consonant span, word span, spatial span SS (visual and auditory) | -.61  .05 | 14 |  |  |
| Sachdev et al., 2006* | 139 | IS and TIA | 72.2 (9.0) | [3-6] | no | no | 100 | both | DSF, DSB | -.41  .01 | 4 |  |  |
| Sachdev et al., 2009 | 104 | IS and TIA | 70.4 (9.2) | [3-6], follow-up + 36 | no | no | 84 | both | DSF, DSB | NA | NA |  |  |
| Saldert et al., 2007* | 14 RH, 14 LH | NS | 62.6 (NS) | > 6 | NS | NS | 14 | low | experimental Reading Span test | -1.01  .08 | 2 |  |  |
| Salis et al., 2018* | 12 aphasia | IS and HS | 54.8 (9.6) | 61 (53) | NS | NS | 7 | low | Word span | -4.10  .65 | 1 |  |  |
| Schaapsmeerders et al., 2013* | 277 | IS | 50.9 (10.3) | 132 (98) | no | NS | 146 | low | PPMST | -.85  .01 | 1 |  |  |
| Schoo et al., 2014* | 40 | IS | 64 (10.7) | < .5 | no | no | 31 | both | DSF, DSB | .50  .06 | 2 |  |  |
| Schweinberger et al., 1992* | 16 LH, 14 RH | IS and HS | 50.2 (11.2) | 19 (NS) | NS | NS | 14 | both | DSF, SDB, SSF, SSB | -.47  .07 | 8 |  |  |
| Selnes et al., 2015 | 27 | IS | 63.7 (NS) | 3 (NS) | NS | no | 41 | high | Letter-number sequencing | NA | NA |  |  |
| Snaphaan et al., 2009* | 28 | IS | 53.7 (12.6) | [1.5 – 3] | no | no | 22 | high | 2-back | -.40  .08 | 1 |  |  |
| Srikanth et al., 2003 | 99 | IS and HS | 70.5 (14.0) | 3 (NS) | no | yes | 99 | both | DST | NA | NA |  |  |
| Stamenova et al., 2017* | 17 | NS | 67.1 (10.8) | 53 (NS) | NS | no | 30 | both | DSF, DSB | .20  .09 | 2 |  |  |
| Stricker et al., 2010 | 42 | NS | 63.2 (10.6) | 96 (75.6) | NS | NS | 36 | both | DSF, DSB | NA | NA |  |  |
| Su et al., 2018* | 25 RH | TIA | 52.4 (4.9) | .25, T2 3 | no | no | 25 | high | 2-back | -.57  .04 | 2 |  |  |
| Van Asselen et al., 2009* | 21 RH, 32 LH, 5 BL | IS and HS | 54.5 (2.9) | > 6 | NS | no | 76 | low | SSF | -.42  .06 | 3 |  |  |
| Van Asselen et al., 2006* | 14 RH, 16 LH | NS | 57.8 (2.9) | > 6 | NS | no | 36 | both | Letter number sequencing, SSF | -2.34  .03 | 4 |  |  |
| Van der Ham et al., 2012 | 16 LH, 17 RH | IS and HS | 59.2 (13.3) | 14 (5) | no | no | 28 | both | Letter number sequencing, SSF, SSB | NA | NA |  |  |
| Van Geldorp et al., 2013* | 24 | IS and HS | 52.1 (11.2) | chronic | NS | NS | 31 | both | DST | -.54  .10 | 2 |  |  |
| Van Zandvoort et al., 2001* | 35 SSLI | IS | 59 (13) | < .5, T2 7 (2) | no | no | 31 | both | DST, SSF | -.62  .03 | 2 |  |  |
| Volle et al., 2008* | 20 | IS and HS | 45.6 (11.3) | 18.3 (16.8) | no | NS | 48 | both | 1-, 2-, 3-back | -.83  .08 | 9 |  |  |
| Weinstein et al., 2014* | 132 | IS and HS | 77.4 (9.4) | 6 (NS) | NS | no | 132 | both | DSF, DSB | -.41  .02 | 2 |  |  |

*Notes.* articles with an asterisk (*) are included in the meta-analyses, other articles are only included in the systematic review. ^1^Interval between stroke and working memory assessment. IS = ischemic stroke; HS = haemorrhagic stroke; LH = left hemisphere; RH = right hemisphere; BL = bilateral; IPC = inferior parietal cortex; IFC = inferior frontal cortex; MCA = middle cerebral artery; AChAI = anterior choroidal artery infarction; SSLI = single supratentorial lacunar infarct; WB = whole brain; NS = not specified; NA = not applicable; DSF = digit span forward; DSB = digit span backward; DST = digit span total; SSF = spatial span forward; SSB = spatial span backward; CDR = Cognitive Drug Research; ADO = ascendant ordering of digits; AS = auditory span of words in sentences; PPMST = Paper and Pencil Memory Scanning Task; ISR = Immediate Serial Recall; PASAT = Paced Auditory Serial Addition Test; TVA = theory of visual attention.

**Table S2**

*RTI items rated for all studies*

|  | **Q1** | **Q2** | **Q3** | **Q5** | **Q6** | **Q7** | **Q8** | **Q9** | **Q13** |
| --- | --- | --- | --- | --- | --- | --- | --- | --- | --- |
| Andrade et al., 2012 | low | low | low | Low | Low | low | N/A | high | high |
| Andrews et al., 2013 | uncl | low | low | uncl | low | high | N/A | low | high |
| Andrews et al., 2014 | uncl | low | low | uncl | low | high | N/A | low | high |
| Baier et al., 2014 | uncl | uncl | uncl | uncl | low | low | N/A | high | high |
| Baldo et al., 2006 | low | P low; HC uncl | low | uncl | low | low | N/A | low | low |
| Beeson et al., 1993 | P low; HC uncl | uncl | low | uncl | low | high | N/A | low | high |
| Bugarski Ignjatovic et al., 2015 | low | P low; HC uncl | low | uncl | low | low | N/A | high | low |
| Burton et al., 2004 | low | low | high | uncl | low | low | N/A | low | high |
| Butler et al., 2009 | uncl | uncl | high | uncl | low | low | N/A | low | high |
| Cao et al., 2007 | P low; HC uncl | P low; HC uncl | low | uncl | low | low | N/A | low | high |
| Danet et al., 2015 | P low; HC uncl | P low; HC uncl | low | uncl | low | low | N/A | low | high |
| Elwan et al., 1994 | low | low | high | uncl | low | high | N/A | low | high |
| Exner et al., 2001 | low | low | low | uncl | low | high | N/A | low | low |
| Fu et al., 2017 | P low; HC uncl | P low; HC uncl | low | uncl | low | low | N/A | high | uncl |
| Godefroy et al., 1994 | P low; HC uncl | low | low | uncl | low | low | N/A | high | uncl |
| Gong et al., 2015 | low | low | low | uncl | uncl | low | N/A | high | high |
| Gorišek et al., 2016 | P low; HC uncl | uncl | uncl | uncl | low | low | N/A | low | high |
| Grosdemange et al., 2015 | low | low | low | high | low | low | N/A | low | low |
| Hirel et al., 2017 | low | P low; HC uncl | low | uncl | low | low | N/A | high | low |
| Ho et al., 2018 | P low; HC uncl | uncl | low | uncl | uncl | low | N/A | high | high |
| Hochstenbach et al., 1998a | low | low | low | uncl | low | high | N/A | low | low |
| Hochstenbach et al., 1998b | low | low | low | uncl | low | high | N/A | high | low |
| Jokinen et al., 2006 | low | low | high | uncl | low | uncl | N/A | high | high |
| Kant et al., 2014 | low | low | low | uncl | low | low | N/A | low | high |
| Karimian et al., 2018 | low | P low; HC uncl | high | high | low | high | N/A | high | low |
| Kessels et al., 2002 | P low; HC uncl | low | low | uncl | low | low | N/A | high | high |
| Kober et al., 2015 | P low; HC uncl | uncl | high | uncl | uncl | high | N/A | high | high |
| Kokubo et al., 2015 | P low; HC uncl | low | low | low | low | low | N/A | low | high |
| Kraft et al., 2015 | low | P low; HC uncl | high | uncl | low | high | N/A | high | high |
| Leskelä et al., 1999 | P low; HC uncl | low | high | low | low | low | N/A | low | high |
| Li et al., 2013 | low | uncl | low | uncl | low | low | N/A | high | high |
| Li et al., 2019 | low | low | low | uncl | low | low | N/A | low | high |
| Liebermann et al., 2013 | low | low | low | uncl | low | high | N/A | high | high |
| Low et al., 2016 | low | low | high | uncl | low | high | N/A | low | high |
| Luukkainen-Markkula et al., 2011 | uncl | P low; HC uncl | low | uncl | low | high | N/A | low | high |
| Malhotra et al., 2005 | uncl | low | high | uncl | low | high | N/A | high | high |
| Malm et al., 1998 | uncl | P low; HC uncl | high | uncl | low | uncl | low | high | high |
| Malouin et al., 2004 | P low; HC uncl | uncl | high | uncl | uncl | low | N/A | high | high |
| Mansueti et al., 2008 | low | low | low | uncl | high | uncl | N/A | low | low |
| Martin et al., 2019 | P low; HC uncl | P low; HC uncl | uncl | uncl | low | low | N/A | low | high |
| Martins et al., 2009 | low | low | low | uncl | low | uncl | N/A | high | high |
| McDonnell et al., 2011 | low | low | low | high | low | low | N/A | high | high |
| Nys et al., 2006 | low | P low; HC uncl | high | uncl | uncl | low | N/A | low | low |
| Oliveira et al., 2015 | low | uncl | low | uncl | uncl | uncl | N/A | high | low |
| Pergola et al., 2012 | low | P low; HC uncl | low | uncl | low | low | N/A | low | low |
| Piccardi et al., 2016 | low | P low; HC uncl | low | uncl | uncl | high | N/A | high | uncl |
| Planton et al., 2012 | low | low | low | uncl | low | low | N/A | high | low |
| Pluta et al., 2017 | P low; HC uncl | uncl | low | uncl | uncl | high | N/A | high | low |
| Ramsey et al., 2017 | low | P low; HC uncl | low | uncl | low | low | low | high | low |
| Rapport et al., 1994 | uncl | uncl | low | uncl | uncl | high | N/A | low | high |
| Ravizza et al., 2005 | uncl | uncl | low | uncl | low | uncl | N/A | high | high |
| Rochat et al., 2013 | P low; HC uncl | low | low | uncl | low | low | low | low | high |
| Rosemann et al., 2017 | low | P low; HC uncl | high | uncl | uncl | low | N/A | low | high |
| Rousseaux et al., 2008 | P low; HC uncl | low | low | uncl | low | low | N/A | high | high |
| Roussel et al., 2012 | P low; HC uncl | P low; HC uncl | low | uncl | low | low | N/A | low | high |
| Sachdev et al., 2006 | low | low | high | uncl | low | low | low | high | low |
| Sachdev et al., 2009 | low | low | high | uncl | low | low | low | high | low |
| Saldert et al., 2007 | low | P low; HC uncl | low | uncl | low | low | N/A | high | high |
| Salis et al., 2018 | uncl | uncl | high | uncl | uncl | low | N/A | low | high |
| Schaapsmeerders et al., 2013 | low | low | low | uncl | low | low | low | low | low |
| Schoo et al., 2014 | low | P low; HC uncl | low | uncl | low | low | N/A | high | low |
| Schweinberger et al., 1992 | P low; HC uncl | P low; HC uncl | low | uncl | low | high | N/A | low | high |
| Selnes et al., 2015 | P low; HC uncl | low | high | uncl | uncl | uncl | N/A | high | high |
| Snaphaan et al., 2009 | P low; HC uncl | P low; HC uncl | low | uncl | low | low | N/A | low | high |
| Srikanth et al., 2003 | P low; HC uncl | low | low | low | low | uncl | N/A | high | high |
| Stamenova et al., 2017 | low | low | low | uncl | uncl | low | N/A | low | high |
| Stricker et al., 2010 | low | P low; HC uncl | low | uncl | low | low | N/A | high | high |
| Su et al., 2018 | low | unclear | low | uncl | low | low | low | low | low |
| Van Asselen et al., 2009 | low | low | low | uncl | low | low | N/A | low | low |
| Van Asselen et al., 2006 | P low; HC uncl | low | low | uncl | uncl | low | N/A | low | high |
| Van der Ham et al., 2012 | P low; HC uncl | P low; HC uncl | low | uncl | low | low | N/A | low | high |
| Van Geldorp et al., 2013 | uncl | P low; HC uncl | low | uncl | uncl | uncl | N/A | high | high |
| Van Zandvoort et al., 2001 | P low; HC uncl | P low; HC uncl | low | uncl | low | low | N/A | low | high |
| Volle et al., 2008 | low | P low; HC uncl | low | uncl | low | high | N/A | low | high |
| Weinstein et al., 2014 | low | low | low | uncl | low | low | N/A | low | high |

*Notes.* P = patient; HC = healthy control; uncl = unclear or not reported; N/A = not applicable

**Table S3a**

*Results of the meta-analyses after exclusion of studies that included TIA patients.*

|  | *k* | *N P/HC* | *ES (g)* | *95% CI* | *Q* | *p (Q)* | *I^2^* | τ*^2^* | *Fail-safe N* | |
| --- | --- | --- | --- | --- | --- | --- | --- | --- | --- | --- |
| Overall | 46 | 2,750/2,506 | -.68 | -.85 to -.52 | 285.35 | <.001 | 84.23 | .25 | 4,202 |  |
| Low-load | 38 | 2,550/2,087 | -.59 | -.79 to -.40 | 308.01 | <.001 | 87.99 | .31 | 2,234 |  |
| High - load | 37 | 2,141/1,916 | -.62 | -.78 to -.46 | 171.10 | <.001 | 78.96 | .18 | 2,231 |  |
| Sub - actute | 12 | 759/395 | -.44 | -.72 to -.16 | 44.78 | <.001 | 75.44 | .18 | 104 |  |
| Chronic | 21 | 1,026/1,117 | -.93 | -1.20 to -.67 | 146.33 | <.001 | 86.33 | .31 | 1,587 |  |

*Notes.* *k* = number of studies; P = patients; HC = healthy controls

**Table S3b**

*Results of the meta-analyses based on span tasks only.*

|  | *k* | *N P/HC* | *ES (g)* | *95% CI* | *Q* | *p (Q)* | *I^2^* | τ*^2^* | *Fail-safe N* | |
| --- | --- | --- | --- | --- | --- | --- | --- | --- | --- | --- |
| Overall | 31 | 2,155/1,805 | -.61 | -.81 to -.40 | 231.68 | <.001 | 87.05 | .28 | 1,578 |  |
| Low- load | 30 | 2,201/1,787 | -.55 | -.76 to -.34 | 235.23 | <.001 | 87.67 | .28 | 1,278 |  |
| High- load | 26 | 1,883/1,433 | -.52 | -.71 to -.34 | 130,00 | <.001 | 80.77 | .17 | 855 |  |
| Sub-acute | 8 | 656/288 | -.19 | -.48 to .10 | 23.34 | .001 | 70.01 | .12 | 9 |  |
| Chronic | 13 | 622/880 | -.87 | -1.26 to -49 | 129.99 | <.001 | 90.77 | .44 | 570 |  |

*Notes.* *k* = number of studies; P = patients; HC = healthy controls
